# Self harm through foreign bodies ingestion – a rare cause of digestive perforation

**Published:** 2014-03-25

**Authors:** S Petrea, I Brezean

**Affiliations:** "I. Juvara" Surgery Clinic, “Dr .I. Cantacuzino” Hospital, Bucharest

**Keywords:** self-harm, ingestion, foreign bodies

## Abstract

Self-harm is a rare pathology, often seen in psychiatric patients but more frequently in the penitentiary environment. Of the many possible forms of self-harm, foreign bodies (FB) ingestion is by far the most usual in the Romanian prison environment. Our paper aims to present the diagnostic and therapeutic aspects arising as a consequence of digestive tract perforations consequent upon foreign bodies ingestion; we analyze a number of 45 cases which occurred over a 7-year period (2003-2010) in Rahova Penitentiary Hospital. We also examined the surgical particularities of case resolution.

Abbreviations: FB – foreign bodies, EEA – end-to-end anastomosis

## Introduction

Self-harm is usually a non-fatal act by which a person deliberately provokes a lesion or ingests a substance in excess to any therapeutic dose or medical prescription. The act must be non-accidental, but may occur without any clear indication of whether the death wish was present or not [**1**]. This type of pathology is prevalent in psychiatric patients, but is also often found amongst penitentiary inmates, though the latter is seldom analyzed or presented in the medical literature.

Self-aggression in the Romanian prison environment is usually caused by either psychiatric pathology or occurs as a form of protest against prison injustice, aimed at obtaining improved conditions for the prisoner or other benefits. It is often perceived as a way of manifesting one’s freedom to act upon one’s own body. 

 In the surgery service of the Rahova Penitentiary Hospital a total of 762 self-harm cases were admitted, diagnosed and treated over a 7-year period (2003-2010).

 472 of these cases were of self-harm by foreign bodies (FB) ingestion. Not all FB ingestions resulted in surgical intervention. Natural elimination processes occurred in 199 (42%) of patients; endoscopic extraction was practiced on 74 (15,69%) cases; 34 patients refused treatment and were not subject to any medical intervention (7,2%). Surgical extraction was chosen in 165 (34,95%) cases and was due to either the failure of endoscopic extraction maneuvers with FB persistence inside the digestive tract or to digestive perforations in 45 (9.4%) cases. These cases of FB ingestions complicated with digestive tract perforations are the subject of our present paper.

## Material and method

Material and method

 Our aim is to present a retrospective study of 45 cases of digestive lesions consequent upon FB ingestion, admitted and treated between 08.2003-02.2010 in Rahova Penitentiary Hospital.

 Analytical criteria were 1. general patient data; 2. surgical history linked to FB ingestion; 3. data about the FB; 4. time elapsed from self-aggression until surgery; 5. clinical diagnosis; 6. laboratory data; 7. digestive perforation; 8. FB properties; 9. surgical procedure; 10. post-operative evolution

 Now follows an analysis of digestive tract perforations as a consequence of FB ingestion, using data presented below.

## Results

1. Age, sex: the age-group distribution of patients with digestive tract perforations consequent upon FB ingestion shows an increased incidence amongst thirty to forty year olds (20 cases) and forty to fifty year olds (17 cases), and a significant decrease in prisoners aged of over fifth decade of age (only 3 cases). The vast majority of the patients treated were men (male/female ratio of 44/1).

 2. Surgical history of FB ingestion: more than half of those presenting with digestive tract lesions due to FB ingestion are first-time surgical patients (24 cases); 40% have a history of 2 to 5 surgical interventions (18 cases); in three cases, the patients were subject to more than 5 interventions.

 3. Data about the FB

 The 45 self-harm cases involved a total of 105 FB; in three quarters of cases just one FB was ingested (33 cases); multiple FB (12 cases) varied between 2 and 12. Most of the FB were metal (metal-only in 40 cases and metal and plastic in 4 cases) comprised of cutlery (35), needles (25), wires (17) and less frequent ingestions were those of nails (7 cases), complete sets of table cutlery (5 cases - two cases each with teaspoons, forks and a knife), nail scissors, a nail clipper, a nail file, a key, a metal rod, and a rolled-up tin can lid. The next most frequent FB were plastic items, including ballpoint pens (5), and toothbrushes (4). We also found one glass FB – a thermometer. 

 FB dimensions were small (0-5 cm) in 17 cases; medium (5-10 cm) in 35 cases; large (10-15 cm) in 40 cases and very large (over 15 cm) in 13 cases. FB edges and extremities were sharp in 54 cases, cutting in 39 cases, blunt in 9 cases, sharp and cutting in 2 cases and sharp and blunt in one case.

 4. Time elapsed between the moment of self-harm and seeking medical care

 Self-harm through FB ingestion is often not followed by the patient asking for medical attention. Almost 2/3 of our patients (31 cases–68%) were seen by a doctor in the first month after the ingestion and in the remainder of the cases the time interval varied between 2 months and over a year. 

 This delay is partly due to the patients themselves, who routinely either hide the self-harm or refuse to be examined by a doctor in the hope of aggravating their lesions and thus obtaining certain advantages by “blackmailing" the penitentiary staff. Sometimes the delay was due to organizational and logistical deficiencies within the penitentiary system (limited possibilities of transport to the penitentiary hospital) and lack of cooperation from other hospitals situated in the area of the penitentiary.

 5. Clinical diagnosis

 The clinic of digestive perforation syndrome due to FB ingestion is subjectively dominated by pain (31 cases) followed by fever. We noted asymptomatic patients in 8 cases, in one of these the FB was partially exteriorized through the orifice of a transcutaneous fistula (gastro-parietal fistula secondary to the ingestion of a 15-cm long wire). In other cases the initial symptoms were due to complications; bloating and intestinal transit stop (intestinal occlusion syndrome - one case); massive hematemesis (gastro - eso - aortic fistula - one case). 

 Objective signs were absent in 60% of cases (27 cases). For the other cases we observed the following, listed in terms of frequency: peritoneal irritation syndrome (11 cases), abdominal palpable mass (4 cases) and a case each of massive hematemesis, abdominal distension, presence of cutaneous fistula through the FB is partially exteriorized.

 Sepsis was present in one case of posterior rectal penetrating FB, with recto-ureteral fistula and retroperitoneal suppuration and in one case of intraduodenal FB with blocked inferior flexure perforation and secondary septic liver and lung determinations.

 6. Paraclinical diagnosis

 Biological diagnosis: in 2/3 of the cases (29 cases) there was no biohumoral modification. Leukocytosis was present in 14 cases as unique laboratory modification; one case associated nitrogen retention; another, with sepsis, presented with anemia, nitrogen retention and hepatic cytolysis syndrome.

 Imaging diagnosis

**Fig. 1:**
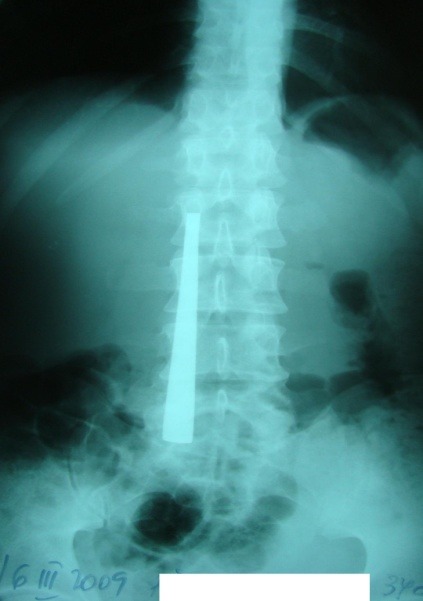
FB (cover handle) situated at the second part of duodenum

 Abdominal X-ray shows radio opaque images of varying intensity, depending on the nature of the FB and its physical characteristics (number, dimension, type of edges and extremities). Follow-up by seriated X-rays every 2 or 3 days may show the progression of the FB through the digestive tract. FB image projection at the level of different abdominal topographic regions generally superimposes over the projections of digestive segments at the same level (epigastrium - gastric FB; mezogastrium - gastric, duodenal, intestinal FB; right hypocondrium - duodenal FB; left hypocondrium - gastric FB; flanks – colonic FB; hypogastrium – intestinal FB). The superimposition of a radiological image over the right edge of the lumbar spine was a marker of a second duodenum positioning of the FB in 10 of the 14 cases (**Fig. 1**).

**Fig. 2:**
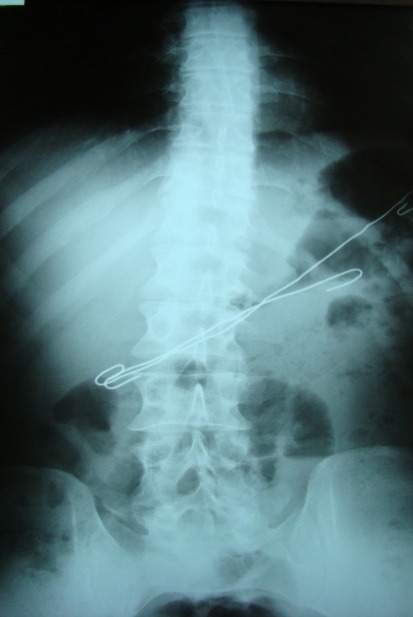
Gastric FB (wires); perforation with peritonitis, hydroaeric levels

**Fig. 3:**
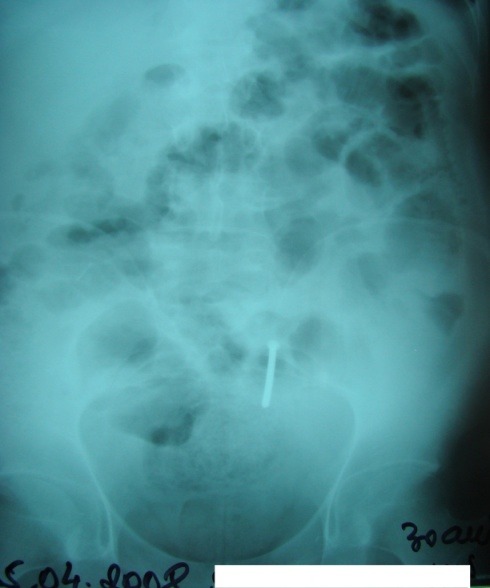
Enteral FB (nail), entero-parietal fistula with occlusive syndrome

 Abdominal X-ray can show images suggesting either FB digestive perforation – as we noted in 2 cases (superior duodenal flexure perforation, recto-ureteral perforation) or intestinal occlusion in 2 cases of generalized peritonitis with secondary ileus (**Fig 2,3**). Pneumoperitoneum, a characteristic of digestive perforations, was only encountered in one case of gastric perforation with neglected secondary peritonitis.

 X-ray with contrast medium (Gastrografin) was used in pinpointing the position of the FB at the gastro-duodenal level in patients refusing superior endoscopic maneuvers.

 Superior endoscopy has not only an important diagnostic role in the case of eso-gastro-duodenal FB, but a curative one as well–one can try extraction. This maneuver is not indicated for patients who present with digestive perforation and signs of peritonitis.

 We used endoscopy for 4 cases. This low number was in part due to impossibility of performing the technique (18 cases), or because the FB had already left the duodenum when we examined the patient (10 cases). In 3 cases the maneuver was not executed due to physical characteristics of the FB (number, size, large weight). The other situations involved the FB leaving the stomach (1 case), patients’ denial (1 case), surgical emergency situations (8 cases).

**Fig. 4:**
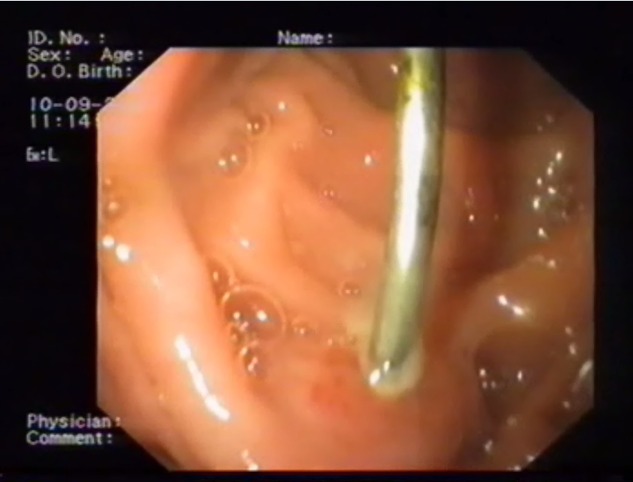
Gastric FB – wire (gastro-jejunal fistula)

**Fig. 5:**
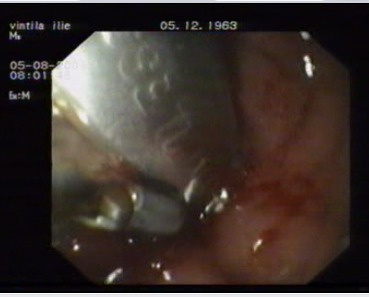
Duodenal FB – cover handle (duodenal-liver penetration)

 FB endoscopic extraction was only successful in one case, in the remaining cases, failure was due to the nature of the FB – large and heavy, as in the case of whole forks and spoons, or to digestive perforation with subsequent FB migration to the adjoining organs (gastric perforation with hepatic penetration in 2 cases and one case of gastric perforation with gastro-jejunal fistula) (**Fig 4,5**).

 We must not minimize the role of technical resources that, if present, might ensure success of endoscopic extraction; crocodile pincers of different sizes overtubes for multiple passages through the eso-gastric junction, the possibility of total anesthesia for the patient.

 Ecography examination was useful in investigating abdominal tumoral masses occurring after FB ingestion, whenever we suspected a digestive perforation with parenchymal organ penetration (liver), when the FB was positioned inside the duodenum, and in the case of FB ingestion associated with pyretic syndrome.

 7. Digestive perforation and FB properties

 Position and number of enteral perforations produced by the FB along the digestive tract were: 1. eso-gastro-aortic fistula – 1 case (2.22%); 2. gastric lesions – 8 cases (17.77%); 3. duodenum lesions – 16 cases (35.55%); 4. enteral lesions – 14 cases (31.11%); 5. colon lesions – 2 cases (4.44%); 6. rectal lesions – 2 cases (4.44%); 7. unknown origin perforations – 2 cases (4.44%)

 FB physical properties and the type of anatomical and clinical lesions it induces in the digestive tract are shown in the next table (**Table 1**).

**Table 1:**
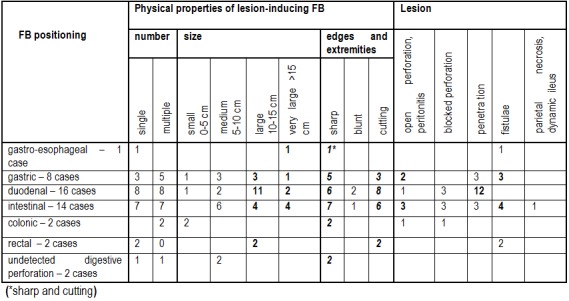
FB physical properties and the type of anatomical and clinical lesions

 We note frequent lesion occurrence in gastric, duodenal and enteral positioning of the FB (38 cases); large and very large FB often cause perforations (28 cases), also sharp and cutting FB (42 cases). The perforative lesion accompanied by peritonitis and fistulae are frequently encountered at both intestinal and gastric levels. When the FB is positioned in the duodenum, we can expect perforations with liver penetration.

 8. Surgical sanction

 Emergency surgery was needed for 8 cases (17.77%), 7 of these cases presented with peritonitis and in one case the FB was exteriorized through a gastro-parietal fistula. In 3 cases (6.66%) although both clinic and imagery justified an emergency surgical intervention, it was delayed due to patient refusal (2 cases of peritonitis, delayed for 2 and 5 days respectively, and one case of knife ingestion with eso-gastro-aortic perforation, delayed for 3 days until a cataclysmic hemorrhage occurred).

 Table 2 shows the types of intraoperative lesions we encountered and their surgical sanction.

**Table 2:**
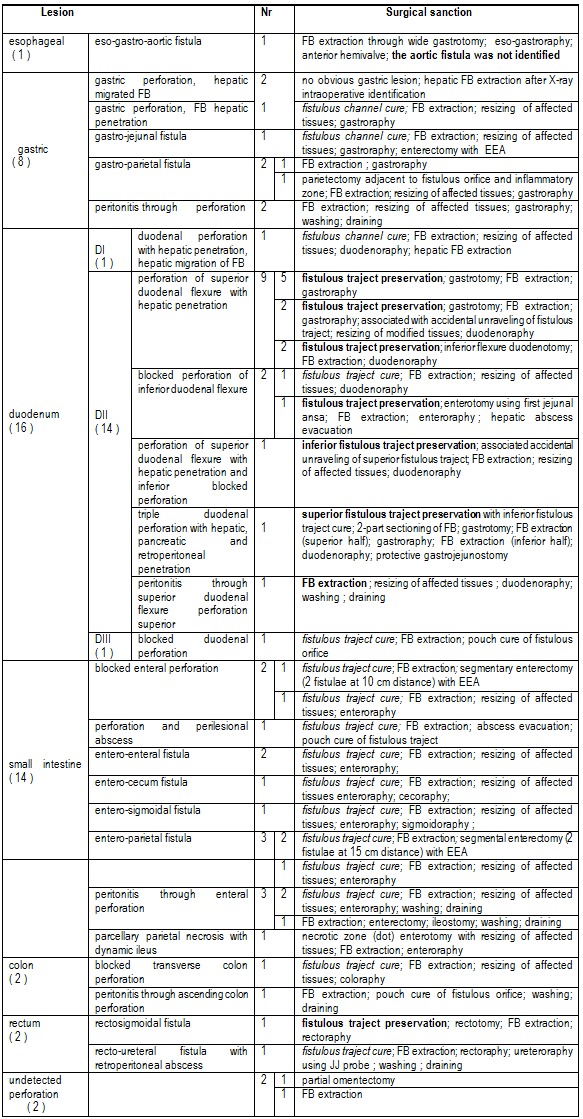
shows the types of intraoperative lesions and their surgical sanction

 9. Post-operative evolution

 Post-operative complications occurred in 10 patients (22.2%).

 Amongst parietal complications, we note 2 eviscerations - a spontaneous, self-caused one, by deliberate extraction of parietal stitches, which was cured by total plan suture and a blocked one, which was treated conservatively.

 Abdominal complications were due to the presence of fistulae. The first, secondary to a gastrotomy, was the consequence of deliberate extraction of parietal stitches and insertion of a FB through the resulting gastrotomy wound, and was treated using conservative methods. The second, a colonic one, needed surgical reintervention. 

 General complications were one case of intraoperative ischemic vascular stroke, and one of hepatic cirrhosis decompensation.

 Death occurred in 3 cases (6.66%). From the total number of patients presenting with self-harm through FB ingestion, this type of evolution was confined exclusively to cases of complicated ingestions. One case exited through hemorrhagic shock due to a fissured post-traumatic aortic aneurysm (the patient with undetected gastro-eso-aortic fistula). The other 2 cases developed acute respiratory distress syndrome due to septic shock lung (one patient with blocked perforation of the inferior duodenal flexure and multiple liver septic abscesses and one patient with enteral blocked perforation and a complex surgical history).

 Discussion

 Taking into account the lack of specialized literature when it comes to self-aggression through FB ingestion, the discussion will confine its scope to the present material. According to medical literature, accidental ingestion of foreign bodies is a not infrequent pathology, but deliberate ingestion is rare, usually found amongst prison inmates and psychiatric patients. In the US 1500 deaths/year are due to accidental FB ingestion (2007) [**2**].

 Our study noted that patients with self-harm through complicated FB ingestion are predominantly male, in the third or fourth decade of life (similar to age and sex distribution found in the penitentiary population) and often do it for the first time.

 Ingested foreign bodies are multiple (53%), metallic (90%), medium or large-sized, sharp (51%), and cutting (37%). 

 In contrast to a British study where the mean time from FB ingestion to seeking qualified medical care was 12 hours (38 patients with accidental and deliberate ingestion of foreign bodies) [**3**], we note that our patients almost never seek qualified medical care in the first days following the ingestion. The vast majority (2/3) see a doctor sometime in the first month after the event, and there were cases in which the interval was around one year. This aspect obviously increases both the number of complications and of surgical interventions.

 A quarter of our patients were admitted in an acute clinical state, the rest of the cases presenting with non-specific or no complaints. 

 Biology is often normal. The principal method in establishing both a clear FB ingestion diagnosis and showing the properties of the FB is X-ray examination. Periodical X-ray exams can pinpoint complications by specific imagery (hydro-aerial levels, pneumoperitoneum, air enteria) or by showing the FB constantly in the same position, which usually indicates a fistula. A persistence of the FB in the duodenum for more than 10 days after ingestion can indicate a fistula – although we also noted it in 2 cases where surgery was performed less than 10 days after ingestion; the same can be said for a persistent, unchanging area of FB projection for more than 4 weeks. 

 Endoscopy can be performed in almost all cases, with the notable exception of perforation associated with peritonitis or penetration in a vascular structure [**4,5**]. Pseudotumoral mass development or transparietal exteriorization of the FB do not represent endoscopic contraindications, but should only play a diagnostic role in such situations. In order to achieve a high success rate when dealing with these cases a number of conditions have to be met simultaneously: 1. such as seeking of medical care quickly (in the literature endoscopic maneuvers were performed in the first 24 to 48 hours after the ingestion); 2. technical and staff capabilities; 3. physical properties (weight, dimensions) which allow the FB to be manipulated; 4. a reasonable number of ingested FB (a limiting number of FB does not exist, if we have the appropriate tools); 5. dexterous practitioner; 6. patient consent. As literature data shows, endoscopy can solve up to 10-20% of uncomplicated FB ingestion cases [**6**]. We found few references to multiple FB ingestion of either medium or small dimensions. In a study of 542 patients with both accidental and deliberate FB ingestion for whom the rate of endoscopic extraction is 19.5%, only 24 patients had ingested foreign bodies larger than 10 cm – and all these cases had a surgical sanction [**7**]. 

 Abdominal ecography can be useful in diagnosing parenchymal penetrations [**8**]. 

 Impactation zones for a FB exiting the esophagus are the pylorus, Treitz’s angle, the ileo-caecal valve, the rectosigmoidal junction. The literature differs when describing the topography of perforations, with a prevalence of ileal and rectocolic lesions and an occurring ratio of peritonitis between 31-44% [**4,6,8-13**]. Although the duodenum is often cited as a preferred impactation zone, it is not considered a complication-prone zone.

 In our study, most of the lesions occurred at duodenal and enteral levels (30 cases-66.6%) and were often blocked (78%), with peritonitis representing 22% of these. Physical properties of the lesion-inducing FB are dependent on their position, 60% of them being larger than 10 cm. For the edges and sides, we note that sharp FB are responsible for gastric and colon fistulae (75% gastric and 100% colonic), cutting FB are the cause of duodenal and rectal lesions (50% duodenal, 100% rectal) and enteral lesions are produced by both FB types in equal measure. Overall though, the proportion inclines towards sharp foreign bodies.

 Our data showed that FB even larger than 10 cm can pass through the duodenum, thus contradicting the literature which states that the maximum dimensions allowing pyloric passage are a diameter of 2 cm and a length of 6 cm (10 cm according to some authors) [**6,14,15**].

 Surgical sanction consisted of direct inter-visceral lesion attack with FB extraction and suturing of perforation-interested segments. In the case of digestive tract lesions with parenchymal organ or abdominal wall penetration, FB extraction was followed by closure of the digestive perforation, while the penetrating trajectory did not require exceptional surgical procedures.

 A special case is the extraction of FB from the duodenum. Our preferred way of access was through the mobile superjacent (stomach) or subjacent (first jejune coil) segments - depending on the FB position. We tried to avoid having to put sutures on a fixed portion of the digestive tube, as this would require special mobilization techniques. Our solution did not produce any complications.

 Our morbidity rate is inferior to that of a 33-case study of accidental ingestions, where it reached a level of 57.6%, but mortalities are comparable [**12**].

 11. Conclusions

 Self-harm is predominantly encountered in the penitentiary environment, FB ingestion being its most frequent manifestation. In contrast to accidental FB ingestion, both the time between ingestion and seeking medical care and the properties of the FB are different in cases of deliberate ingestion, leading to a particular type of lesions.

 Surgical sanction per se does not imply complex operations, but postoperative evolution may be negatively affected by continued self-aggression, either by repeating the FB ingestion, or by deliberate early parietal and even visceral suture ripping.

 Better life conditions inside the penitentiary, perfecting ways of efficient communication between prisoners and staff, education and skill-learning amongst the inmates, innovative, civilized and healthy ways of spending free time, respect of inmate rights according to the law, could all lead, in time, to a decrease in the number of self-aggression cases.

 Disclosures - None
